# Increased Mortality Rates Associated with *Staphylococcus aureus* and Influenza Co-infection, Maryland and Iowa, USA^1^

**DOI:** 10.3201/eid2207.151319

**Published:** 2016-07

**Authors:** Jennifer S. McDanel, Eli N. Perencevich, Jeremy Storm, Daniel J. Diekema, Loreen Herwaldt, J. Kristie Johnson, Patricia L. Winokur, Marin L. Schweizer

**Affiliations:** University of Iowa, Iowa City, Iowa, USA (J.S. McDanel, E.N. Perencevich, J. Storm, D.J. Diekema, L. Herwaldt, P.L. Winokur, M.L. Schweizer);; Iowa City Veterans Affairs Health Care System, Iowa City (J.S. McDanel E.N. Perencevich, P.L. Winokur, M.L. Schweizer);; University of Iowa Hospitals and Clinics, Iowa City (D.J. Diekema, L. Herwaldt);; University of Maryland School of Medicine, Baltimore, Maryland, USA (J.K. Johnson)

**Keywords:** *Staphylococcus* *aureus*, staphylococci, pneumonia, influenza, respiratory infections, viruses, bacteria, mortality rate, co-infection, comorbidity, MRSA

## Abstract

We retrospectively analyzed data for 195 respiratory infection patients who had positive *Staphyloccocus aureus* cultures and who were hospitalized in 2 hospitals in Iowa and Maryland, USA, during 2003–2009. Odds for death for patients who also had influenza-positive test results were >4 times higher than for those who had negative influenza test results.

*Staphylococcus aureus* is a common cause of respiratory infections, including pneumonia (*1*), and can lead to necrotizing pneumonia and death (*2*–*4*). Influenza complicated by *S. aureus* co-infection can progress rapidly to death within a week of symptom onset (*3*,*4*). However, few studies have evaluated whether patients who are co-infected with influenza and *S. aureus* are more likely to experience poor outcomes compared with patients who are infected with *S. aureus* alone. We compared patient characteristics and outcomes of patients who had a respiratory culture that grew *S. aureus* and who tested positive for influenza with those who had negative influenza test results.

## The Study

This retrospective cohort study included pediatric and adult patients admitted to the University of Iowa Hospitals and Clinics (Iowa City, IA, USA) or to the University of Maryland Medical Center (Baltimore, Maryland, USA) during 2003–2009. First, we used codes from the International Classification of Diseases, Ninth Revision, Clinical Modification (ICD-9-CM), to identify patients with influenza-like illness (ILI) (*5*). This criterion was part of an initial study investigating influenza-like illness and *S. aureus* pneumonia (J.S. McDanel, unpub. data). Patients were included in the study if they had respiratory cultures (sputum, bronchial specimen, or tracheal aspirate) that grew *S. aureus* and were tested for influenza before or during their admissions. If a patient was admitted >1 time, only the admission with the first *S. aureus* positive respiratory culture was included. The University of Iowa institutional review board approved this study.

The primary outcome of interest, 30-day in-hospital mortality, was defined as death occurring in the hospital within 30 days of the first culture that grew *S. aureus*. The adapted Charlson Comorbidity Index served as an aggregate score for co-occurring conditions (*6*). The year of each patient’s first positive *S. aureus* culture was dichotomized: 2003–2007 and 2008–2009.

We conducted bivariable analyses using either the χ^2^ test or the Fisher exact test for categorical variables and the Student *t*-test or Wilcoxon rank-sum test for continuous variables. We used logistic regression to identify associations between potential predictor variables and 30-day mortality rates. We included variables in the multivariable model using a manual stepwise method. Variables associated with death (p<0.25) in the bivariable regression analysis were examined for fit within the multivariable model and were retained if statistically significant (p<0.05). The year of each patient’s first positive *S. aureus* culture was forced into the model. We analyzed data using SAS software version 9.3 (SAS Institute, Cary, NC, USA).

A total of 195 patients had >1 respiratory culture that grew *S. aureus* and were also tested for influenza. Sputum samples (115, 59%) and bronchial washes (50, 26%) were the most common respiratory specimens. Blood cultures of 17 (9%) patients grew *S. aureus*. Respiratory or blood samples of 109 (56%) patients grew methicillin-resistant *S. aureus* (MRSA). Most patients (166, 85%) were admitted to the University of Maryland Medical Center; 116 (59%) were male, and median age was 42 (interquartile range 5–59) years. 

Of the 195 patients, 32 (16%) had positive influenza test results. Patients who had a positive influenza test were more likely to receive quinolones (odds ratio [OR] 3.30, 95% CI 1.51–7.21) than were patients whose influenza tests were negative (Table 1). Patients who had a positive influenza test were significantly more likely to have the positive *S. aureus* respiratory culture collected <2 days after hospital admission than were the patients whose influenza tests were negative (OR 3.27, 95% CI 1.39–7.70).

**Table 1:** Characteristics of patients in cohorts demonstrating increased mortality rates associated with *Staphylococcus* *aureus* and influenza co-infection, Maryland and Iowa, USA*

Characteristic	**Positive influenza test, n = 32**	**Negative influenza test, n = 163**	**Odds ratio 95% CI**	**p value**
Female sex	12 (38)	67 (41)	0.86 (0.39–1.88)	0.704
Age ≥18 y	24 (75)	112 (69)	1.37 (0.58–3.25)	0.479
Hospital admission within previous 12 mo	12 (38)	75 (46)	0.70 (0.32–1.53)	0.376
Previous MRSA infection or colonization	4 (13)	33 (20)	0.56 (0.18–1.72)	0.307
Co-occurring conditions				
Cancer	3 (9)	24 (15)	0.60 (0.17–2.12)	0.579
Cerebrovascular disease	0 (0)	6 (4)	UTD	0.592
Chronic pulmonary disease	11 (34)	41 (25)	1.56 (0.69–3.51)	0.282
Heart disease	4 (13)	20 (12)	1.02 (0.32–3.22)	1.000
Diabetes	6 (19)	15 (9)	2.28 (0.81–6.41)	0.123
Liver disease	0 (0)	6 (4)	UTD	0.592
Renal disease	5 (16)	8 (5)	3.59 (1.09–11.79)	0.042
Charlson Comorbidity Index score, median (IQR)	1 (0–2)	1 (0–3)	0.89 (0.75–1.07)	0.641
Methicillin resistance				
MRSA	16 (50)	93 (57)	0.75 (0.35–1.61)	0.462
MSSA	15 (47)	66 (40)	1.30 (0.61–2.78)	0.503
Unknown	1 (3)	4 (2)	1.28 (0.14–11.86)	1.000
First positive *S. aureus* culture collected ≤2 d after hospital admission	24 (75)	78 (48)	3.27 (1.39–7.70)	0.005
Year of first positive *S. aureus* culture				0.038
2003	1 (3)	3 (2)	Reference	
2004	1 (3)	10 (6)	0.30 (0.01–6.38)	
2005	7 (22)	25 (15)	0.84 (0.08–9.38)	
2006	0 (0)	25 (15)	UTD	
2007	2 (6)	14 (9)	0.43 (0.03–6.41)	
2008	12 (38)	26 (16)	1.39 (0.13–14.73)	
2009	9 (28)	60 (37)	0.45 (0.04–4.81)	
Antimicrobial drugs received				
Vancomycin	25 (78)	128 (79)	0.98 (0.39–2.44)	0.960
Linezolid	7 (22)	26 (16)	1.48 (0.58–3.77)	0.414
Quinolone	19 (59)	50 (31)	3.30 (1.51–7.21)	0.002
Macrolide	13 (41)	58 (36)	1.24 (0.57–2.69)	0.588
Aminoglycoside	2 (6)	41 (25)	0.20 (0.05–0.87)	0.018
Cephalosporin	20 (63)	105 (64)	0.92 (0.42–2.02)	0.836
30-d in-hospital deaths	9 (28)	18 (11)	3.15 (1.27–7.86)	0.021
*Values are no. (%) patients except as indicated. MRSA, methicillin-resistant *Staphylococcus aureus*; UTD; unable to determine because calculation includes zero; IQR; interquartile range; MSSA, methicillin-susceptible *S. aureus*.				

Of the 32 influenza-positive patients, 9 (28%) died; of the 163 influenza-negative patients, 18 (11%) died (OR 3.15, 95% CI 1.27–7.86; p = 0.021) (Table 2). Of the 9 influenza-positive patients who died, 5 had MRSA. Among the 27 patients who died, those with a positive influenza test were more likely to have diabetes than those who had a negative influenza test (33% vs. 0%; p = 0.029). The multivariable logistic regression model found that, after statistically adjusting for year and time from admission to collection of *S. aureus* culture samples, patients whose influenza tests were positive had >4-fold increased odds of death compared with patients whose influenza tests were negative (OR 4.31, 95% CI 1.57–11.83; p<0.005) (Table 2).

**Table 2:** Adjusted regression analysis of the association between influenza and 30-d in-hospital deaths among patients with *Staphylococcus aureus*–positive respiratory cultures, Maryland and Iowa, USA*

Model and variable	**Odds ratio (95% CI)**	**p value**
Unadjusted		
Influenza-positive test	3.15 (1.27–7.86)	0.021
Adjusted†		
Influenza-positive test	4.31 (1.57–11.83)	0.005
First positive *S. aureus* culture collected ≤2 d after hospital admission	3.00 (1.18–7.61)	0.021
Year of first positive *S. aureus* culture, 2008–2009†	1.71 (0.70–4.13)	0.237

*Defined as death occurring in the hospital within 30 d of the first respiratory culture that grew *S. aureus*. †Reference 2003–2007.

## Conclusions

Our results are consistent with the results of other studies. Other investigators reported poor outcomes among patients who were co-infected with influenza viruses and *S. aureus* (*3*,*4*,*7*). Kallen et al. found a statistically significant increased risk for death among patients who had positive influenza test results and community-acquired *S. aureus* pneumonia, compared with patients who had negative influenza test results and community-acquired *S. aureus* pneumonia (*7*). The Kallen et al. study included patients who had either MRSA or methicillin–susceptible *S. aureus* pneumonia (*7*) but evaluated only 47 patients. The sample size for our study was much larger than previously performed studies, and we were able to examine mortality rates among patients who had a respiratory culture that grew either MRSA or methicillin-susceptible *S*. *aureus*.

Additionally, co-infection with influenza and *S. aureus* has been examined in animal models to identify mechanisms that cause poor outcomes (*8*–*12*). Severity of illness related to co-infection has been associated with a dysfunctional cell repair system and an altered immunologic response such as suppression of macrophage function, inhibition in phagocytic bacterial clearance, and cell damage to the airway system (*8*–*12*). Investigators have hypothesized that influenza damages epithelial cells in the respiratory system, providing opportunity for enhanced bacterial attachment (*8*,*11*). Once bacteria invade, cell destruction and fluid cause dysfunction of the airway system (*8*,*11*).

This study had limitations. First, the investigation might have excluded patients who were tested for influenza at other facilities or who did not have laboratory-confirmed influenza. Second, we could not determine whether the respiratory cultures that grew *S. aureus* represented infections or colonization. However, the information we describe remains clinically relevant because often clinicians do not know whether patients with positive *S. aureus* cultures are infected or colonized. Diagnosing *S. aureus* pneumonia is challenging, and acquiring a lower respiratory culture such as a bronchial specimen or tracheal aspirate can be invasive and difficult to collect. Therefore, if *S. aureus* pneumonia is suspected (e.g., symptoms and positive sputum culture), patients may be treated without a confirmed positive lower respiratory culture. Third, our dataset did not include information about variables such as influenza vaccination status, mechanical ventilation, co-infection with organisms other than influenza and *S. aureus*, and whether the pneumonia was necrotizing. Fourth, misclassification bias may exist based on our definition of influenza infection. Patients with a negative influenza test may be misclassified since we were unable to determine the time interval between the onset of ILI symptoms and the collection of the influenza sample. Therefore, patients may have recovered from influenza before receiving an influenza test. Last, influenza-like illness ICD-9-CM codes were used to identify the cohort because the patients initially were included in a study of influenza-like illness and *S. aureus* pneumonia (J.S. McDanel, unpub. data). Therefore, patients may have been missed if they had a respiratory infection with *S. aureus* and the condition or symptoms were not captured through an ICD-9-CM code.

In conclusion, among patients whose respiratory cultures grew *S. aureus*, patients with influenza were significantly more likely to die than were patients whose influenza tests were negative. Interventions that increase influenza vaccination rates among patients at high risk for *S. aureus* respiratory infections may prevent both co-infection and death.
